# Structural Features of Antitumor Titanium Agents and Related Compounds

**DOI:** 10.1155/BCA.2005.317

**Published:** 2005

**Authors:** Francesco Caruso, Miriam Rossi, Cristian Opazo, Claudio Pettinari

**Affiliations:** 1 Instituto di Chimica Biomolecolare CNR Piazzale Aldo Moro, 5 Rome 00185 Italy; 2 Vassar College Department of Chemistry Poughkeepsie NY 12604-0484 USA; 3 Vassar College SCIVIS laboratory Poughkeepsie NY 12604-0724 USA; 4 Università di Camerino Facoltà di Scienze Chimiche Via S. Agostino 1 Camerino (MC) Italy

## Abstract

Previous studies established some Ti compounds as having marked activity against tumors of the gastrointestinal tract and lack of side effects common to widely used cytostatic agents. We describe pertinent structural features of known antitumor Ti agents and other potentially active compounds. Particularly noteworthy features are that Ti-O bonds are short and Ti-O-Ti bond angles are large, demonstrating that in these compounds the O binding has high s-character approaching sp hybridization.


### Structural Features of Antitumor Titanium Agents and Related Compounds

Francesco Caruso,*[,a] Miriam Rossi[b], Cristian Opazo[c] and Claudio Pettinari[d]

[a]*Instituto di Chimica Biomolecolare, CNR,Piazzale Aldo Moro, 5, 00185, Rome, Italy.*
[b]*Vassar College, Department of Chemistry, Poughkeepsie, NY, 12604-0484, USA*
[c]*Vassar College, SCIVIS laboratory, Poughkeepsie, NY, 12604-0724, USA*
[d]*Università di Camerino, Facoltà di Scienze Chimiche; Via S. Agostino 1, Camerino (MC) Italy*

### ABSTRACT

Previous studies established some Ti compounds as having marked activity against tumors of the gastrointestinal tract and lack of side effects common to widely used cytostatic agents. We describe pertinent structural features of known antitumor Ti agents and other potentially active compounds. Particularly noteworthy features are that Ti-O bonds are short and Ti-O-Ti bond angles are large, demonstrating that in these compounds the O binding has high s-character approaching sp hybridization.

---

The successful drug development /1/ of the antitumor agent *cis*-diaminodichloroplatinum(II) (cisplatin) **1**, generated a search for other active metal compounds and *cis*-diethoxy-bis(1-phenylbutane-1,3-dionato)titanium(IV), $[(bzac)_2Ti(OEt)_2]$ (budotitane) **2**, was the first non-Pt metal antitumor compound that reached clinical trials /2/. The ligand 1-phenylbutane-1,3-dionato = bzac = benzoylacetonato, is an asymmetric β-diketone chelator useful for establishing one key structural feature for activity in budotitane, namely, the existence of two OEt *cis* leaving groups, which are analogous to the 2 Cl in cisplatin. Another Ti antitumor agent is titanocene dichloride, $(Cp)_2TiCl_2$ **3**, Cp = cyclopentadienyl, which possesses 2 Cl leaving groups /5/.

Among the differences between Ti antitumor drugs and cisplatin is the spectrum of activity, as Ti drugs operate against gastrointestinal tumors whereas Pt drugs do not. On the other hand, P338 and L1210 leukemia are sensitive targets for Pt drugs but not for budotitane /4/. Other differences include a much faster hydrolysis rate of the leaving groups in the former, and the environment where the metal-leaving group bonds cleave: outside the cell for Ti drugs and inside the cell for Pt drugs /1/.

Titanocene dichloride shows a larger spectrum of activity compared to budotitane. This is likely due to better solubility in physiological medium and it is currently in phase II clinical tests /5/, whereas the

* E-mail francesco.caruso@icb.cnr.it, Fax (39) 06 49913628

development of budotitane is limited by formulation problems /6/. Titanocene dichloride interacts with transferrin, a protein associated with iron transport, and suggests a possible mode of entry into the tumor cell. That is, the protein, with a Ti atom bound to one of its 2 domains /7,8/, could cross the tumor cell wall, which is characterized by greater amount of transferrin receptors than present in normal cells, and allow metal interaction with unknown targets.

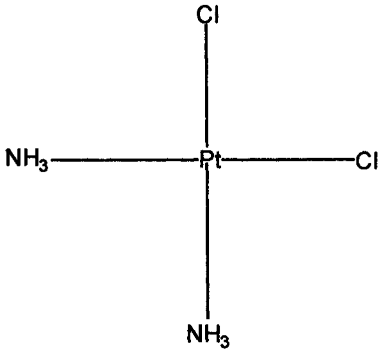

**Fig. 1:** Cisplatin 1.

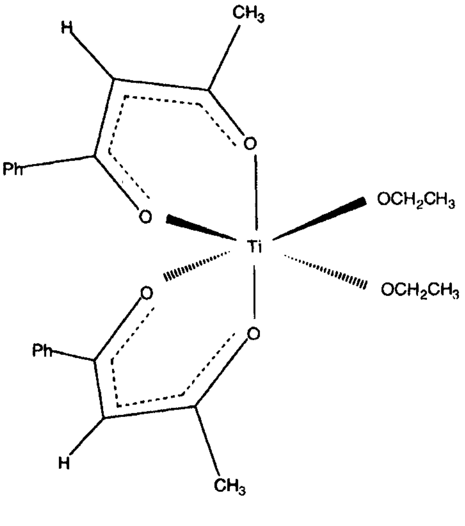

**Fig. 2:** A budotitane stereoisomer 2.

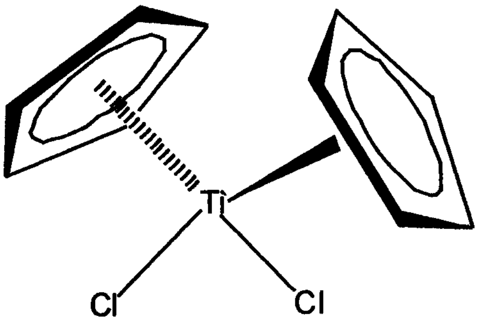

**Fig. 3:** Titanocene dichloride 3.

Due to fast hydrolysis of Cl in titanocene dichloride and OEt in budotitane, oligomerization of Ti compounds is seen. Two of these products are also active, $[(Cp)_2TiCl]_2(\mu\text{-}O)$ **4** /9/ and $[(bzac)_2TiCl]_2(\mu\text{-}O)$ **5** /10/.

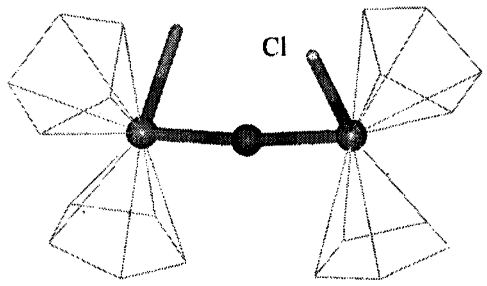

**Fig. 4:** X-ray structure of $[(Cp)_2TiCl]_2(\mu\text{-}O)$ **4**, Ti-O-Ti bond angle = 174°.

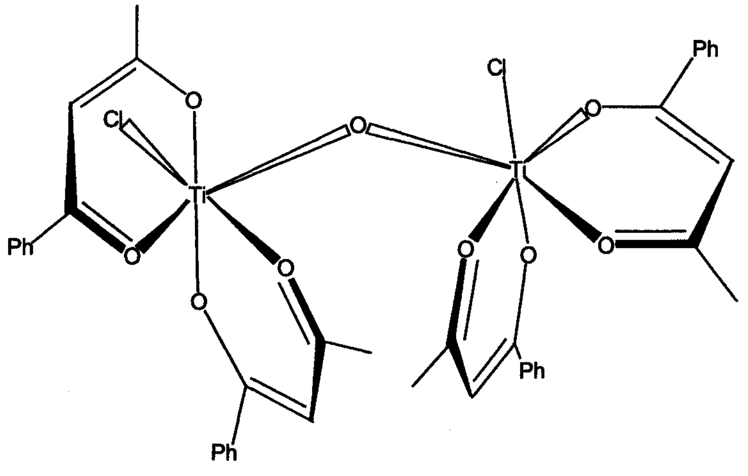

**Fig. 5:** $[(bzac)_2TiCl]_2(\mu\text{-}O)$ **5**.

Since β-diketone asymmetry is an essential feature for high activity in budotitane and related compounds /2/, we are studying closely related Ti derivatives with 4-acyl-5-pyrazolones **6** as ligands, see Figure 6. These are asymmetric β-diketones having 3 possible substitution options.

A compound showing *in vivo* antitumor activity (T/C about 300%) against TA-3 (mouse mammary adenocarcinoma) in AJ mice, slightly lower than that found for budotitane against other tumors /2/, was synthesized by our group /11/. It is a bis(4-acyl-5-pyrazolonato)titanium(IV) tetranuclear cyclic species, $[(Q^B)_2Ti(\mu\text{-}O)]_4$ 7; $Q^B$ has $R^1 = R^4 =$ Ph, $R^3 =$ Me, see Figure 7. Compound 7 is a stable intermediate in the Ti-monomer hydrolysis/polymerization pathway that interfered with budotitane studies, where hydrolysis of OEt and bzac ultimately can induce formation of $TiO_2$.

Since ligand asymmetry is an unexplained yet relevant parameter in budotitane studies we describe selected features of related compounds. A tetranuclear species $[(tmhd)_2Ti(\mu\text{-}O)]_4$ **8**, containing the symmetric

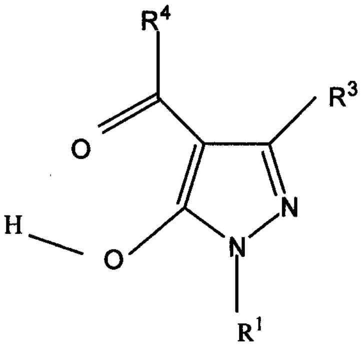

**Fig. 6:** 4-Acyl-5-pyrazolone keto-enol form, HQ **6**.

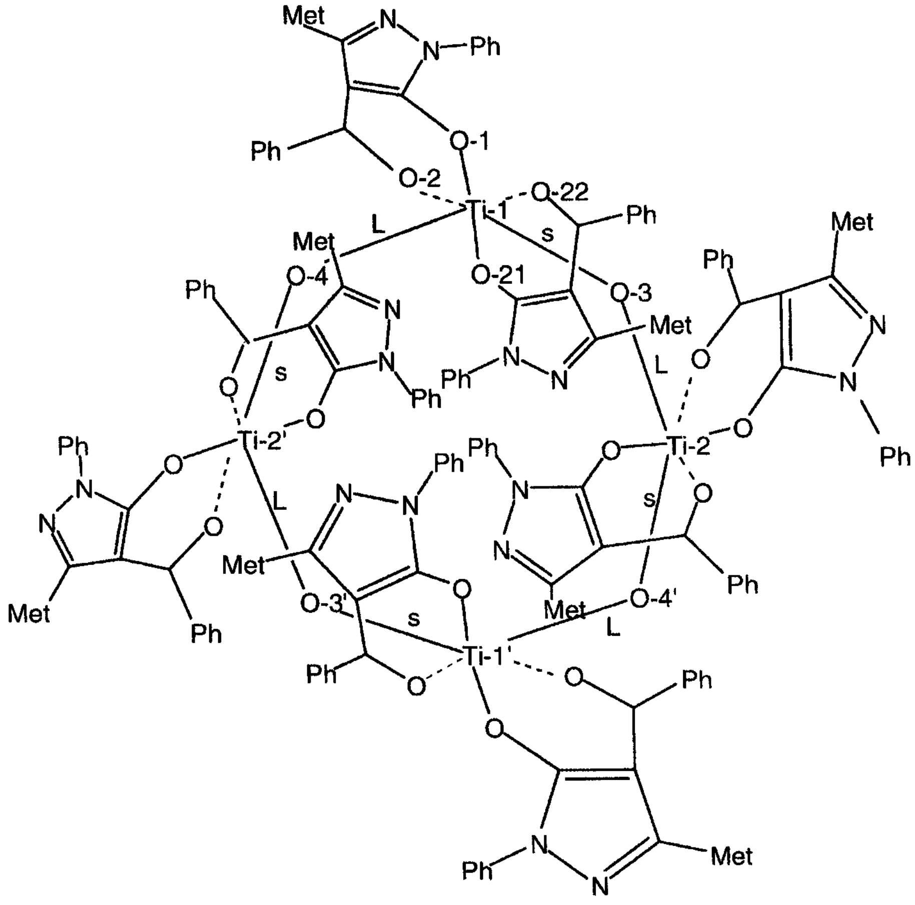

**(a)**

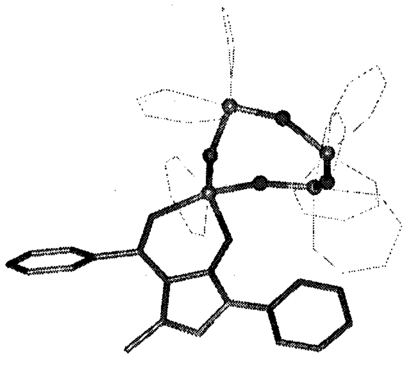

(b)

**Fig. 7:** Tetranuclear 4-acyl-5-pyrazolonato-Ti species $[(Q^B)_2Ti(\mu\text{-}O)]_4$ **7**, ($Q^B$: $R^1 = R^4$ = Ph, $R^3$ = Me). (a) Scheme showing short (s) and long (L) Ti-O bonds in the 8-membered ring and dashed long Ti-O(acyl) bonds; (b) X-ray structure showing the non planar core complex, enolato rings and one whole ligand.

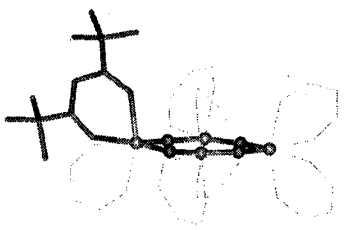

**Fig. 8:** X-ray structure of $[(tmhd)_2Ti(\mu\text{-}O)]_4$ **8**. The symmetric ligand tmhd induces equal Ti-O bond lengths in the planar 8-membered ring. The core complex, enolato rings, and one whole ligand are shown.

β-diketonato ligand tmhd = 2,2,6,6-tetramethylheptane-3,5-dionato, shows 3 sets of Ti-O bond distances /12/, see Figure 8. Set (a) describes the shortest bonds that are located in the 8-membered ring (Ti-μ-O bonds), with values about 1.81 Å. Set (b) includes the longest bonds (about 2.12 Å) that are *trans* to the Ti-O bonds forming set (a). Set (c) shows the intermediate Ti bonds that are *trans* to each other (about 1.97 Å); sets (b) and (c) are formed by Ti-O(tmhd) bonds. The driving force responsible for these relevant geometrical features is the *trans* influence, a property of some metals in their coordination complexes.

From the X-ray crystal structure of **7** there is further splitting of sets (a) and (b) and an alternate sequence of short-long lengths in the Ti-μ-O bonds ($Ti_1$-$O_3$ = 1.767(6) Å, $O_3$-$Ti_2$ = 1.859(6) Å, $Ti_2$-$O_{4'}$ = 1.758(5) Å, $O_4$-$Ti_{1'}$ = 1.868(4) Å), see Figure 7a. *Trans* to each Ti-μ-O bond a Ti-O(acyl) bond is found; these are also split. The 8-membered ring common to **7** and **8** is further affected by ligand asymmetry, as the latter is planar whereas the former is not. In addition, Ti-O-Ti bond angles are about 170° in **8** whereas in **7** they are about 150°.

As already mentioned, dinuclear compounds **4** and **5** are also active. Therefore the tendency of Ti to polymerize, through O(oxo) bridges, raises the question as to whether a polynuclear form is an entity needed to induce antitumor activity, as seen for compound **7.** We also analyze structurally related Ti polynuclear species, not yet tested, indicating the parent antitumor compound they stem from, if applicable. Dinuclear compounds include, $[(Cp)_2Ti(H_2O)]_2(\mu\text{-}O)]^{2+}$ **9**, obtained from $(Cp)_2TiCl_2$ /13/ and $[(Cp)TiCl_2]_2(\mu\text{-}O)$ **10** /14/ obtained from $(Cp)TiCl_3$. Some trinuclear complexes include $[(Cp)_3Ti_3(\mu_3\text{-}O)(\mu\text{-}OH)_3(\mu\text{-}HCOO)_3]^+$ **11**, a cationic cyclic species obtained after hydrolysis of $(Cp)_2TiCl_2$ and addition of formic acid /15/; the cationic cyclic species $[(Cp)_3Ti_3(\mu_3\text{-}O)(\mu\text{-}OMe)_3(OMe)_3]^+$ **12**, /16/; $[(Cp)_2TiCl(\mu\text{-}O)]_2(Cp)TiCl]^{3+}$ **13**, a non-cyclic species obtained from $(Cp)_2TiCl_2$ hydrolysis /17/ and the neutral species $[(Me_5\text{-}Cp)_2TiCl(\mu\text{-}O)]_3$ **14** /18/. Compounds **11** and **13** are obtained after Ti-Cp cleavage. This is an important step in titanocene dichloride mechanism of action because it is required for Ti interaction with transferrin. Some tetranuclear compounds are $[(Br(CH_2)_2\text{-}Cp)Ti(\mu\text{-}O)Br]_4$ /19/ and $[(Me_5\text{-}Cp)Ti(\mu\text{-}O)Br]_4$ **15** /20/. A tetranuclear species with additional O(oxo) bridges is $[(Me_5\text{-}Cp)_4Ti_4(\mu\text{-}O)_5Cl_2]$ **16** /21/, whereas $[(Me_4Ph\text{-}Cp)_4Ti_4(\mu\text{-}O)_6]$ **17** shows a later stage of Cl hydrolysis /22/. Even more complex structures are cluster compounds, such as $[(Cp)_6Ti_6(\mu_3\text{-}O)_6(\mu_3\text{-}Cl)_2]$ **18** /23/ and a cage example is $[(Cp)_8Ti_8(\mu\text{-}O)_{12}]$ **19** /24/.

Compounds **15-17** are tetranuclear Ti–Cp derivatives closely related to those of β-diketone ligands of Figures 7 and 8. Further, compounds **4**, **7-10**, **12**, **13**, **15** and **19** show large Ti-O(oxo)-Ti bond angles and short Ti-O(oxo) bond lengths. The large values of Ti-O-Ti angles are not induced by steric hindrance. For instance, the μ-O(oxo) atom links 2 $TiCpCl_2$ groups in compound **10** with a Ti-O(oxo)-Ti bond angle of 180° and two Ti-O(oxo) of 1.73 Å, whereas, in compound **4** it links 2 bulkier $TiCp_2Cl$ groups with a Ti-O(oxo)-Ti bond angle of 174° and Ti-O(oxo) of 1.83 Å.

The evolution of Ti-Cp compounds towards $TiO_2$ through hydrolysis can be seen by following the sequence of figures 3, 4, 9, 14-19, that is, loss of Cl, formation of Ti-O-Ti bridges and loss of Cp. The same trend is seen for Ti-β-diketone compounds in the order 2, 5, 7, 8. This behavior suggests that titanocene dichloride and budotitane share similar biological pathways. Compounds **9-19** could aid in clarifying the mechanism of Ti antitumor action if appropriate formulations become available.

Mononuclear derivatives $Q_2TiX_2$, X = Cl, OMe, OPr, were recently also obtained /25/. NMR shows features consistent with hydrolysis; for instance, in $(Q^B)_2Ti(OPr^n)_2$ formation of $Pr^nOH$ is detected after 20 minutes. Hydrolysis is reversible since upon addition of $Pr^iOH$, formation of ($Ti\text{-}OPr^i$)-containing species is observed. This reverse hydrolysis is related to that obtained when EtOH is added to polynuclear species stemming from previous budotitane hydrolysis /2/. We describe a hydrolysis step of the bis-β-diketonato titanium species, $(acac)_2Ti(OH)(OCH_3)$, acac = acetylacetonato, as studied by Density Functional Theory (DFT).

$$(acac)_2Ti(OH)(OCH_3) + H_2O \longrightarrow (acac)_2Ti(OH)_2 + CH_3OH$$

The energy of the products is 15 Kcal/mol higher than that of the reagents. Nonetheless, this feature agrees with the reverse hydrolysis of Ti-OEt and Ti-OPr bonds mentioned above. Therefore, the direction of hydrolysis is likely determined by environmental conditions such as acidity and reagent/product concentration. This may be related to the fast reaction of titanocene dichoride with proteins in the body /26/.

Octahedral 6-coordinate titanium derivatives containing asymmetric ligands can theoretically exist in solution as a mixture of several *cis* and *trans* isomers. However, for related dialkoxy- and dihalo-

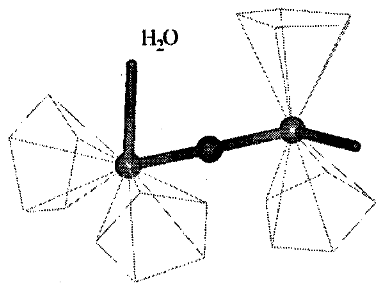

**Fig. 9:** X-ray structure of $[(Cp)_2Ti(H_2O)]_2(\mu\text{-}O)]^{2+}$ **9**, Ti-O-Ti bond angle = 177°.

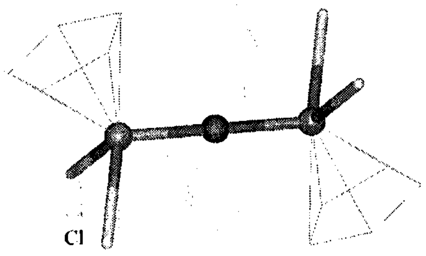

**Fig. 10:** X-ray structure of $[(Cp)TiCl_2]_2(\mu\text{-}O)$ **10**, Ti-O-Ti bond angle = 180°.

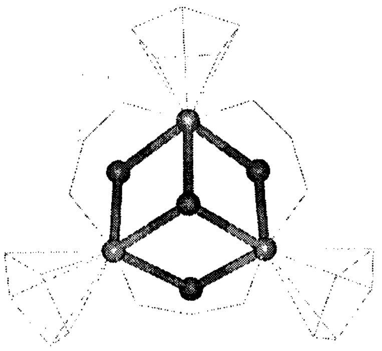

**Fig. 11:** X-ray structure of $[(Cp)_3Ti_3(\mu_3\text{-}O)(\mu\text{-}OH)_3(\mu\text{-}HCOO)_3]^+$ **11**.

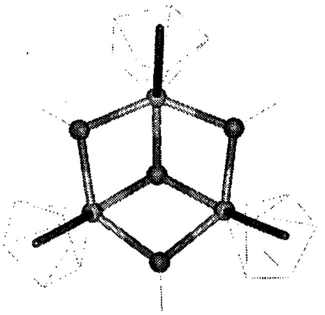

**Fig. 12:** X-ray structure of $[(Cp)_3Ti_3(\mu_3\text{-}O)(\mu\text{-}OMe)_3(OMe)_3]^+$ **12**.

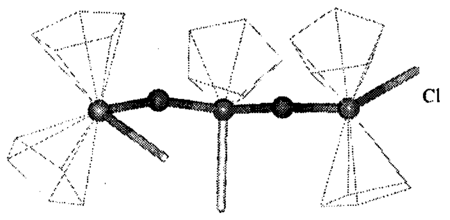

**Fig. 13:** X-ray structure of $[(Cp)_2TiCl(\mu\text{-}O)]_2(Cp)TiCl]^{3+}$ **13**, Ti-O-Ti bond angles = 162° and 176°.

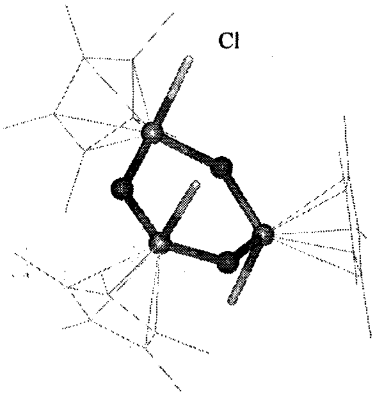

**Fig. 14:** X-ray structure of $[(Me_5\text{-}Cp)TiCl(\mu\text{-}O)]_3$ **14**.

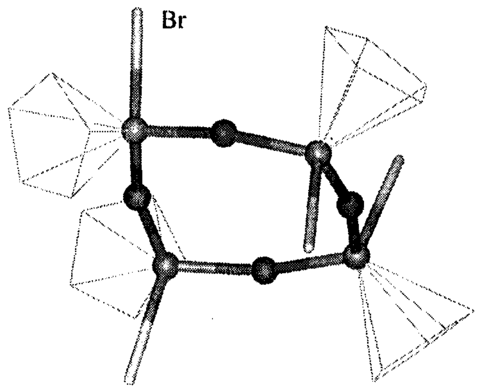

**Fig. 15:** X-ray structure of $[(Me_5\text{-}Cp)Ti(\mu\text{-}O)Br]_4$ **15**, Cp substituents omitted. Ti-O-Ti bond angles = 161°-164°.

bis(acetylacetonato)titanium(IV) derivatives, *cis* forms are strongly preferred /27/. We calculate minimum energy geometry using DFT methods for the three *cis* stereoisomers of $Q_2TiX_2$ (CIS1, CIS2 and CIS3). For $(Q^T)_2TiCl_2$ ($Q^T$: $R^1$ = Ph, $R^3$ = Me, $R^4$ = neopentyl) these *cis* conformers have equivalent calculated energies (within 2.2 Kcal/mol), see Figures 20–22, which agrees with budotitane isomer interconversion shown by NMR /2/. Also $(Q^B)_2Ti(OMe)_2$ conformers show similar energies (within 2.8 Kcal/mol). In contrast, the CIS1

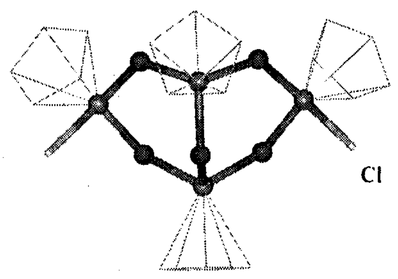

**Fig. 16:** X-ray structure of $[(Me_5\text{-}Cp)_4Ti_4(\mu\text{-}O)_5Cl_2]$ **16**, Ti-O-Ti bond angle = 122° and 128°; Cp substituents omitted.

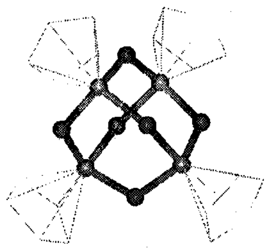

**Fig. 17:** X-ray structure of $[(Me_4Ph\text{-}Cp)_4Ti_4(\mu\text{-}O)_6]$ **17**, Ti-O-Ti bond angles = 96°-129°; Cp substituents omitted.

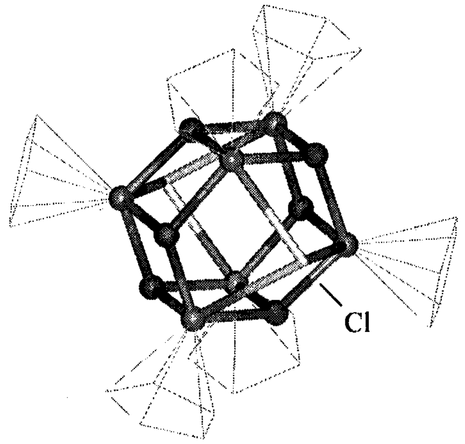

**Fig. 18:** X-ray structure of $[(Cp)_6Ti_6(\mu_3\text{-}O)_6(\mu_3\text{-}Cl)_2]$, **18**.

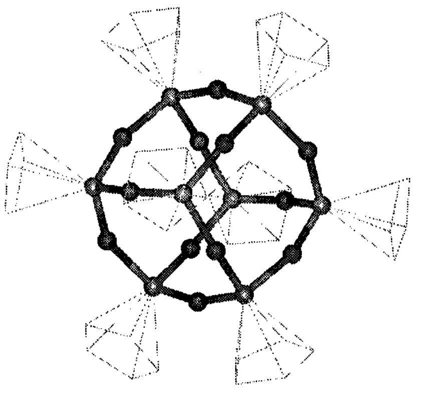

**Fig. 19:** X-ray structure of $[(Cp)_8Ti_8(\mu\text{-}O)_{12}]$ **19**. Ti-O-Ti bond angle = 152°-161°.

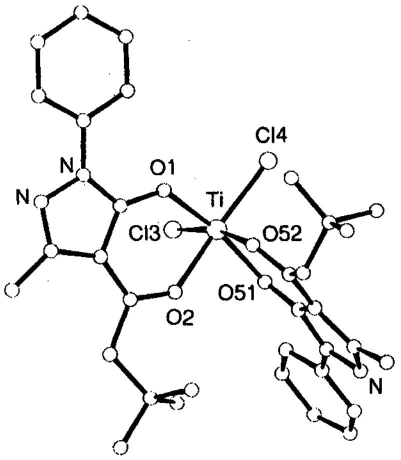

**Fig. 20:** DFT structure of the $(Q^T)_2TiCl_2$ conformer CIS1.

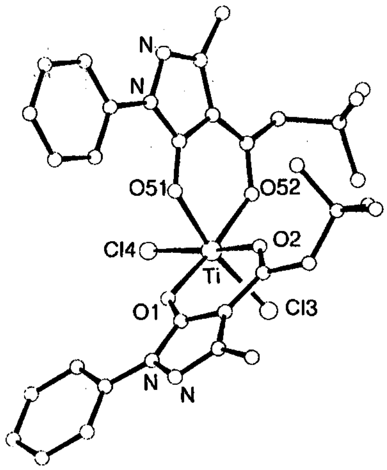

**Fig. 21:** DFT structure of the $(Q^T)_2TiCl_2$ conformer CIS2.

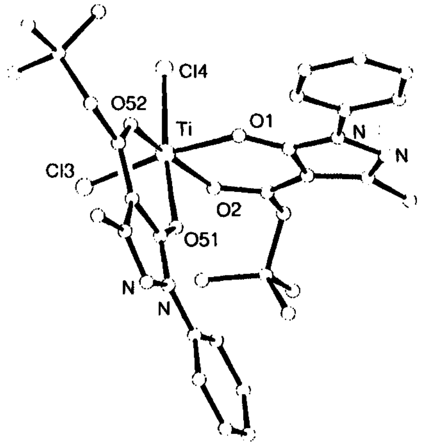

**Fig. 22:** DFT structure of the $(Q^T)_2TiCl_2$ conformer CIS3.

conformer of $(Q^T)_2Ti(OMe)_2$ has markedly higher energy (56 Kcal/mol) than CIS2 and CIS3, likely due to the bulky $R^4$ substituent neopentyl.

In both $(Q^B)_2Ti(OMe)_2$ and $(Q^T)_2Ti(OMe)_2$ structures, the O(methoxy) atoms form the shortest bonds and their opposite bonds are weakened. This *trans* influence is coherent with that experimentally observed for O(oxo) atoms in $[(Q^B{}_2)Ti(\mu\text{-}O)]_4$ /28/. For $(Q^T)_2Ti(OMe)_2$ (CIS3) these data correspond to the pair of opposite bonds Ti-O3 = 1.801 Å, Ti-O1 = 2.084 Å, and Ti-O4 = 1.816 Å, Ti-O51 = 2.046 Å. Completing the coordination sphere are 2 O(acyl) atoms opposite each other; here there is no dominant *trans* influence and both Ti-O(acyl) bonds are equal (Ti-O2 = 1.994 Å, Ti-O52 = 1.992 Å). For the Cl derivative a lengthening of the Ti-Cl bond is expected due to the larger covalent radius /28/ of Cl (0.99 Å) compared with that of O (0.73 Å) in methoxy monomers. However, calculated values of about 0.42 Å overshadow the difference between Cl and O radii (0.26 Å). A partial double bond character of the Ti-O(methoxy) bonds in $Q_2Ti(OCH_3)_2$ explains this feature and the consequent stronger Ti-chelate bonds induced in $(Q^T)_2TiCl_2$, see Table 1.

**Table 1**

Selected structural parameters of bis(4-acyl-5-pyrazolon-5-ato)titanium conformers obtained with DFT methods, and related polynuclear Ti-β-diketonato compounds, obtained with diffraction methods.

| Conformer | Ti-Y3[b] | Ti-Y4[b] | Ti-O1 | Ti-O2[c] | Ti-O51 | Ti-O52[c] | Y-Ti-Y[b] | Ti ligands |
|---|---|---|---|---|---|---|---|---|
| energy[a] | | | 1st Chelate | | 2nd Chelate | | | |
| CIS1 | 2.235 | 2.233 | 1.952 | 2.038 | 1.966 | 2.027 | 102.6 | $Q^T$ |
| −3512.92248 | Y = Cl | Y = Cl | | | | | Y = Cl | Cl |
| CIS2 | 2.233 | 2.236 | 2.021 | 1.968 | 1.955 | 2.048 | 102.3 | $Q^T$ |
| −3512,91900 | Y = Cl | Y = Cl | | | | | Y = Cl | Cl |
| CIS3 | 2.234 | 2.237 | 2.041 | 1.950 | 2.047 | 1.957 | 98.8 | $Q^T$ |
| −3512.91935 | Y = Cl | Y = Cl | | | | | Y = Cl | Cl |
| CIS3 | 1.797 | 1.801 | 2.085 | 2.012 | 2.042 | 2.010 | 95.0 | $Q^B$ |
| −2892.38076 | Y = O | Y = O | | | | | Y = O | $CH_3O$ |
| CIS3 | 1.801 | 1.816 | 2.084 | 1.994 | 2.046 | 1.992 | 95.1 | $Q^T$ |
| −2823.92115 | Y = O | Y = O | | | | | Y = O | $CH_3O$ |
| Polynuclear Compounds | Ti-O3 | Ti-O4 | Ti-O1 | Ti-O2 | Ti-O51 | Ti-O52 | O-Ti-O[d] | Ti-O-Ti[e] |
| | | | 1st Chelate | | 2nd Chelate | | | |
| $[(Q^B)_2Ti(\mu\text{-}O)]_4$[f] | 1.767 | 1.868 | 1.979 | 2.146 | 1.980 | 2.056 | 99.2 | 150.5 153.8 |
| 2nd Ti unit | 1.758 | 1.859 | 1.983 | 2.163 | 1.978 | 2.076 | 100.2 | 150.5 153.8 |
| $[L_2Ti(\mu\text{-}O)]_4$[g] | 1.804 | 1.818 | 1.969 | 2.116 | 1.969 | 2.117 | 100.5 | 170.3 169.3 |
| 2nd Ti unit | 1.810 | 1.810 | 1.968 | 2.120 | 1.968 | 2.120 | 100.3 | 170.3 169.3 |
| 3rd unit | 1.793 | 1.793 | 1.969 | 2.132 | 1.969 | 2.132 | 99.4 | 170.3 169.3 |
| $[(L')_2Ti(\mu\text{-}O)]_2$[h] | 1.831 | 1.824 | 1.968 | 2.059 | 1.974 | 2.059 | 83.4 | 97.1 97.1 |

[a] Energy units (Hartrees) for geometrically converged conformers.

[b] Y3 and Y4 are: O associated to methoxy groups in $(Q^T)_2Ti(OCH_3)_2$ and $(Q^B)_2Ti(OCH_3)_2$; Cl in $(Q^T)_2TiCl_2$; $Q^B$ = 1-phenyl-3-methyl-4-benzoyl-pyrazolon-5-ato, $Q^T$ = 1-phenyl-3-methyl-4-neo-pentyl-carbonyl-pyrazolon-5-ato. In the corresponding columns for the polynuclear compounds Ti-O(oxo) bonds are shown.

[c] O1 and O2 belong to the 1st β-diketonato chelate; O51 and O52 belong to the 2nd β-diketonato chelate.

[d] This parameter is O3-Ti-O4.

[e] These parameters are Ti-O3-Ti and Ti-O4-Ti.

[f] $[(Q^B)_2Ti(\mu\text{-}O)]_4$ has an inversion center that makes two Ti units equivalent to the other two [11]. Its conformation is similar to CIS1.

[g] $[L_2Ti(\mu\text{-}O)]_4$ has a 2-fold axis passing on two Ti atoms; L = 2,2,6,6-tetramethylheptane-3,5-dionato = tmhd [12].

[h] The 2nd Ti unit is crystallographically related by an inversion center; L' = acetylacetonato [27].

Therefore, the *trans* influence provides the driving force for structural features in titanium 4-acyl-5-pyrazolonates. The oxygen atoms O(oxo) in $[(Q^B)_2Ti(\mu\text{-}O)]_4$, and O(methoxy) in $Q_2Ti(CH_3O)_2$, Q = $Q^T$ and $Q^B$, are strongly bound to Ti, with the shortest Ti-O bonds. This results in the lengthening of their opposite Ti-O bonds in the coordination sphere.

### CONCLUSIONS

The role of asymmetry in octahedral antitumor Ti β-diketonates may be related to some weak bonds that such compounds have, like the Ti-O(acyl) bond in $[(Q^B)_2Ti(\mu\text{-}O)]_4$ or those opposed to Ti-O(methoxy) in $Q_2Ti(OMe)_2$. Their cleavage may favor formation of stronger Ti-electrophile bonds, or induce hydrolysis, etc. Such interactions with transferrin may be critical to activity. Novel Ti drug development may come from the investigation of polynuclear species as suggested by active related derivatives of budotitane and titanocene dichloride, and the more recent $[(Q^B)_2Ti(\mu\text{-}O)]_4$. Several polynuclear Ti derivatives here analyzed show markedly short Ti-O(oxo) bonds and large Ti-O(oxo)-Ti bond angles resulting from high s-character of the O(oxo) binding. The short Ti-O(methoxy) bonds in mononuclear methoxy derivatives behave similarly. Useful formulations for these poorly water-soluble species are required; such an approach was successful for $[(Q^B)_2Ti(\mu\text{-}O)]_4$ through a liposome /11/.

### REFERENCES

1. P. Pil and S. Lippard, In: *Encyclopedia of Cancer*; J. R. Bertino (Ed.), Academic Press, San Diego USA, 1997, 392-410.
2. B. K. Keppler, C. Friesen, H. Vongerichten and E. Vogel, In: *Metal Complexes in Cancer Chemotherapy*; B. K. Keppler (Ed.), VCH, Weinheim, 1993, 297-323.
3. M. M. Harding and G. Mokdsi, *Curr. Med. Chem.* 7, 1289 (2000).

4. B. K. Keppler, C. Friesen, H. G. Moritz, H. Vongerichten and E. Vogel, *Struct. Bond.*, **78**, 97 (1991).
5. G. Lümmen, H. Sperling, H. Lubolt, T. Otto and H. Rübben, *Cancer Chemother. Pharmacol.*, **42**, 415 (1998).
6. T. B. Schilling, B. K. Keppler, M. E. Heim, G. Niebch, H. Dietzfelbinger, J. Rastetter and A. R. Hanauske, *Invest. New Drugs*, **13**, 327 (1996).
7. M. Guo, H. Sun, H. J. McArdle, L. Gambling and P. J. Sadler, *Biochemistry*, **39**, 10023 (2000);
8. L. Messori, P. Orioli, V. Banholzer, I. Pais and P. Zatta, *FEBS Letters*, **442**, 157 (1999).
9. Y. Le Page, J. D. McCowan, B. K. Hunter and R. D. Heyding, *J. Organomet. Chem.*, **193**, 201 (1980).
10. P. Koepf-Maier, S. Grabowski and H. Koepf, *Eur. J. Med. Chem.*, **19**, 347 (1984).
11. F. Caruso, M. Rossi, J. Tanski, R. Sartori, R. Sariego, S. Moya, S Diez, E. Navarrete, A. Cingolani, F. Marchetti and C. Pettinari, *J. Med. Chem.*, **43**, 3665 (2000).
12. S. I. Troyanov and O. Y. Gorbenko, *Polyhedron*, **16**, 777 (1997).
13. U. Thewalt and B. Kebbel, *J. Organomet. Chem.*, **150**, 59 (1978).
14. P. Corradini and G. Allegra, *J. Amer. Chem. Soc.*, **81**, 5510 (1959).
15. K. Doppert and U. Thewalt, *J. Organomet. Chem.*, **301**, 41 (1986).
16. H. Aslan, T. Sielisch and R. D. Fischer, *J. Organomet. Chem.*, **315**, C69 (1986).
17. H. P. Klein, U. Thewalt K. Doppert and R. Sanchez-Delgado, *J. Organomet. Chem.*, **236**, 189 (1982).
18. T. Carofiglio, C. Floriani, A. Sgamellotti, M. Rosi, A. Chiesi-Villa and C.Rizzoli, *J. Chem. Soc. Dalton Trans.*, **1992**, 1081.
19. Z. Li, J. Huang, Y. Qian, A. S-C. Chan, K. S-Y. Leung and W. T. Wong, *Inorg. Chem. Comm.*, **2**, 396 (1999).
20. F. Palacios, P. Royo, R. Serrano, J. L. Balcazar, I. Fonseca and F. Florencio, *J. Organomet. Chem.*, **375**, 51 (1989).
21. P. Yu, T. Pape, I. Uson, M. A. Said, H. W. Roesky, M. L. Montero, H-G. Schmidt and A. Demsar, *Inorg. Chem.*, **37**, 5117 (1998).
22. M. Bjoergvinsson, S. Halldorsson, I. Arnason, J. Magull and D. Fenske, *J. Organomet. Chem.*, **544**, 207 (1997).
23. A. Roth, C. Floriani, A. Chiesi-Villa and C. Guastini, *J. Amer. Chem. Soc.*, **108**, 6823 (1986).
24. F. Heshmatpour, S. Wocadlo, W. Massa, K. Dehnicke, F. Bottomley and R. W. Day, *Zeit. Natur. B*, **49**, 827 (1994).
25. F. Caruso, L. Massa, A.Gindulyte, C. Pettinari, F. Marchetti, R. Pettinari, M. Ricciutelli, J. Costamagna, J.C. Canales, J. Tanski and M. Rossi, *Europ. J. Inorg. Chem.*, **2003**, 3221.
26. H. Wittrisch, H-P. Schroeder, J. Vogt and C. Vogt, *Electrophoresis*, **19**, 3012 (1998).
27. G. D. Smith, C. N. Caughlan and J. A. Campbell, *Inorg. Chem.*, **11**, 2989 (1972).
28. L. Pauling, *The Nature of the Chemical Bond*, Cornell University Press, Ithaca, NY, 3rd ed., 1960, 260.

